# Chromosome-level assembly of *Dermatophagoides farinae* genome and transcriptome reveals two novel allergens Der f 37 and Der f 39

**DOI:** 10.1016/j.waojou.2021.100590

**Published:** 2021-09-28

**Authors:** Jiajie Chen, Zelang Cai, Dingding Fan, Jiayu Hu, Yibo Hou, Yongsen He, Zhen Zhang, Zhenfu Zhao, Pan Gao, Wanzhen Hu, Jinlyu Sun, Jiang Li, Kunmei Ji

**Affiliations:** a Department of Biochemistry and Molecular Biology, Laboratory Department of Pinghu Hospital, Health Science Center, Shenzhen University, Shenzhen, 518060, China; b EasyATCG L.L.C, Shenzhen, 518000, China; c Shenzhen University General Hospital, Shenzhen, 518060, China; d Allergy Department, State Key Laboratory of Complex Severe and Rare Diseases, Peking Union Medical College Hospital, Chinese Academy of Medical Sciences & Peking Union Medical College, Beijing, 100730, China

**Keywords:** *Dermatophagoides farinae*, Dust mite, Chromsome-scale genome, Nanopore sequencing, Transcriptome sequencing, Der f 37, Der f 39

## Abstract

Accurate house dust mite (HDM) genome and transcriptome data would promote our understanding of HDM allergens. We sought to assemble chromosome-level genome and precise transcriptome profiling of *Dermatophagoides farinae* and identify novel allergens. In this study, genetic material extracted from HDM bodies and eggs were sequenced. Short-reads from next generation sequencing (NGS) and long-reads from PacBio/Nanopore sequencing were used to construct the *D. farinae* nuclear genome, transcriptome, and mitochondrial genome. The candidate homologs were screened through aligning our assembled transcriptome data with amino acid sequences in the WHO/IUIS database. Our results showed that compared with the *D. farinae* draft genome, bacterial DNA content in the presently developed sequencing reads was dramatically reduced (from 22.9888% to 1.5585%), genome size was corrected (from 53.55 Mb to 58.77 Mb), and the contig N50 was increased (from 8.54 kb to 9365.49 kb). The assembled genome has 10 contigs with minimal microbial contamination, 33 canonical allergens and 2 novel allergens. Eight homologs (≥50% homology) were cloned; 2 bound HDM allergic-sera and were identified as allergens (Der f 37 and Der f 39). In conclusion, a chromosome-level genome, transcriptome and mitochondrial genome of *D. farinae* was generated to support allergen identification and development of diagnostics and immunotherapeutic vaccines.

Letter to the editor,

House dust mites (HDMs), especially *Dermatophagoides pteronyssinus* (Der p) and *Dermatophagoides farinae* (Der f), are major sources of inhaled allergens.^1^, ^2^, ^3^, ^4^ In-depth analyses of the full spectrum of HDM allergens are needed to elucidate HDM allergy mechanisms and guide diagnostic and immunotherapeutic development.^5^

Publication of a Der f draft genome produced with next generation sequencing (NGS) facilitated the identification of numerous allergens,^6^, ^7^, ^8^, ^9^, ^10^, ^11^ designated by the World Health Organization/International Union of Immunological Societies Allergen Nomenclature Sub-committee (WHO/IUIS).^12^ However, the Der f draft genome has shortcomings due to technical limitations.^5^ For example, the Der f 23 cDNA sequence differs from its draft genome corollary.^13^ Because microbiota sequences were removed manually, the draft may contain microbiome sequences.^5^ Additionally, limitations of short-read sequencing were likely to produce much scaffold gaps.^5^

During genome assembly, it is important to minimize cross-species DNA contamination.^14^ Herein, we conducted DNA sequencing of HDM eggs with little microbial genetic contamination. Long-read sequencing with PacBio and Nanopore was performed to obtain a chromosome-level assembly. Homology comparison was performed to optimize transcriptome accuracy. Novel HDM allergen candidates were evaluated with specific immunoglobulin (Ig)E-binding assays.

Due to the symbiotic relationship in the digestive tract, it was not possible to obtain pure mite bodies without any microorganisms through aseptic culture methods.^5^ Our attempts to isolate Der f cells aseptically were not successful (data not shown). To minimize microbial DNA contamination, we isolated HDM eggs (Fig S1A, B) by centrifugation with a density gradient solution and extracted genomic DNA for short-read sequencing. A library was constructed from 500-base pair (bp) fragments, generating a total of 43.7 Gb of data (Table S1). Read assignment analysis showed that the bacterial content was reduced by 93% in reads obtained from eggs (1.5585%) compared to that from bodies (22.9888%). The main bacterial species contaminating body reads were *Proteobacteria* (19.24%), *Bacteroidetes* (2.31%), and *Firmicutes* (0.78%). The main species in the egg reads were also *Proteobacteria* (0.58%), *Bacteroidetes* (0.63%) and *Firmicutes* (0.18%) (Fig S1C, D). The eukaryotic read content was improved by 27% (eggs, 98.3437% vs. bodies, 76.8943%; FIG S1E, F). These data indicate that sequencing reads obtained from HDM eggs constituted a good template for genome assembly.

To reduce gaps, 25.7 Gb of raw long-read sequencing data were obtained from Nanopore sequencing with 400-fold coverage of the estimated genome size. The N50 length of the raw Nanopore reads was 23.9 kb (Table S2). Because long reads may contain errors, assembly data (∼8.3 Gb) were obtained by filtering and correcting ∼22.7 Gb of raw read data (Table S2). Our assembly strategy is summarized in Fig. S2. Using read data from Der f-egg genomic DNA as a template, with an identity criterion of ≥90%, we obtained an assembly with no large gaps and an average sequence depth that had half of the genome sequence coverage as hybrid sequences. We removed 6 hybrid sequences that totaled 104,483 bp.

The assembled genome was submitted to the National Center for Biotechnology Information (BioProject ID PRJNA512594; accession no. SDOV00000000). Based on the updated assembly, the Der f genome size was corrected from 53.55 Mb to 58.77 Mb, with 10 contigs (Table S3). Contig N50 was increased from 8.54 kb to 9365.49 kb, and the contig N90 quantity was decreased from 6350 to 8 (Table S4). The final genome obtained with Nanopore sequencing consists of 10 contigs and a circular mitochondrial DNA (Fig. 1). With the exception of Contig2, all sequences exceeded 2 Mb, with the longest one exceeding 13 Mb, indicating that the assembly quality reached a near-chromosome level (Table S5).

**Fig. 1:**
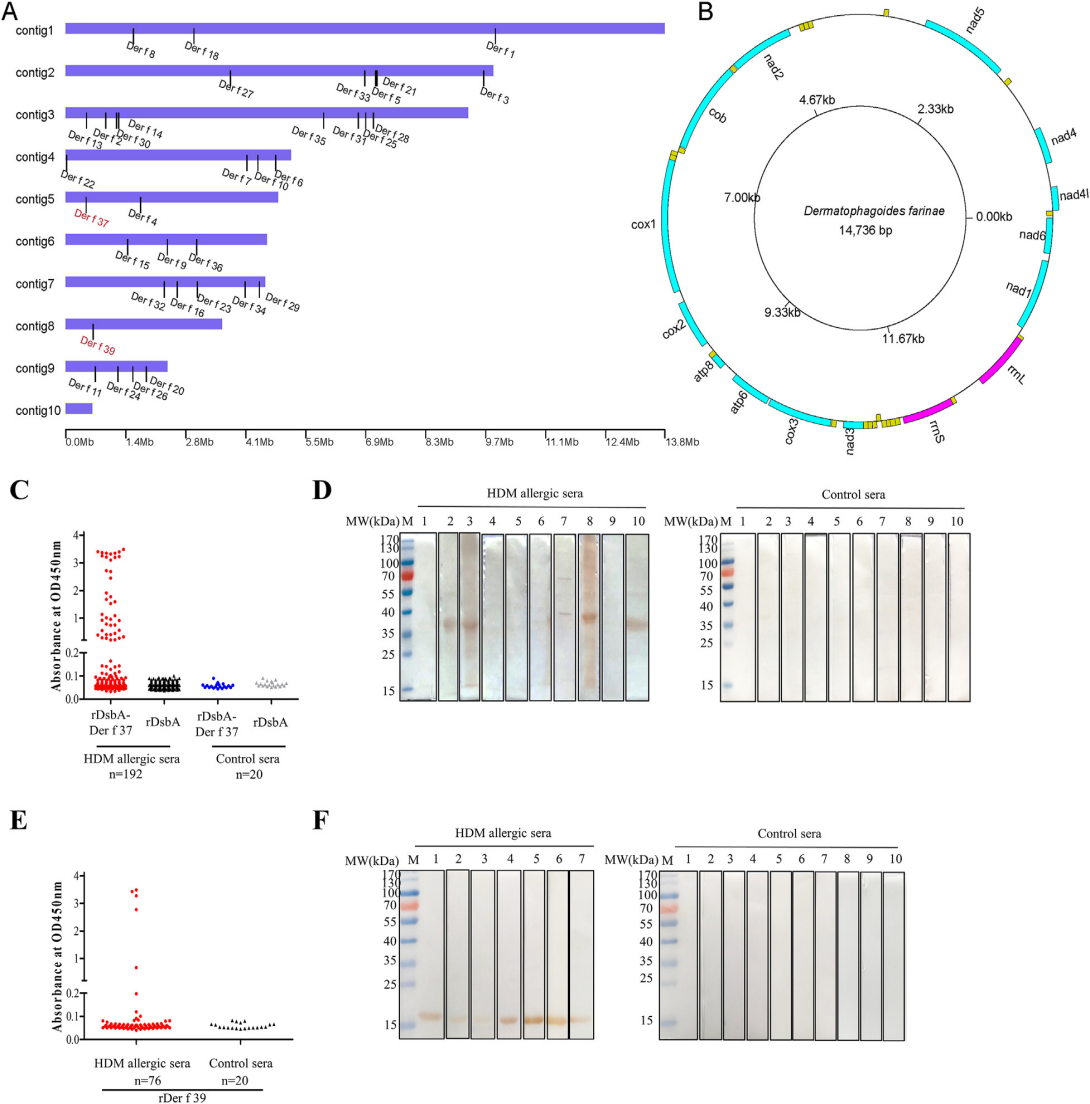
Chromsome-level assembly of genome and mitochondrion genome of *D. farinae* and identification of novel HDM allergens Der f 37 and Der f 39. **A.** Genome assembly showing locations of allergen genes. **B.** Mitochondrial genome of *D. farinae.* Mite allergens based on WHO/IUIS allergen nomenclature (http://www.allergen.org/search.php?TaxSource=Animalia%20Arthropoda). Der f 17 sequence information: not available; HDM allergen Groups 12 and 19: not found; Der p 38 homolog in *D. farinae* (bacterial lytic enzyme like protein, GenBank accession No. MT360919.1) showed no IgE-binding activity in this study. **C.** IgE binding activity determined by IgE-western blots of rDsbA-Der f 37 with individual sera from 192 HDM-allergic patients and 20 healthy non-allergic individuals. **D.** Western blot assay identifying rDer f 37 protein binding by IgE in sera from 10 patients with HDM allergies (left) and 10 non-HDM allergic subjects (control, right). **E**. IgE binding activity determined by IgE-ELISA of rDer f 39 with individual sera from 76 HDM-allergic patients and 20 healthy non-allergic individuals. **F.** Western blot assay identifying rDer f 39 protein binding by IgE in sera from 7 patients with HDM allergies (left) and 10 non-allergic subjects (control, right). The HDM-specific IgEs (>100 kU_A_/L) within the sera samples were evaluated using an ImmunoCAP system.

To construct an allergen gene map (Fig. 1A), we annotated 33 canonical allergen genes in the assembled genome, including 2 newly discovered proteins, namely Der f 37 and Der f 39, in corresponding contig positions. To obtain a high-quality gene set, we performed homology, next generation RNA sequencing (RNA-seq), and *de novo*-based genome annotation of the chromosome-level assembly. For RNA-seq, we obtained 10.66 Gb of RNA-seq reads from mite bodies and 41,602 transcripts from a PacBio Iso-Seq assembly with an N50 size of 2627 (Table S6). We identified 10,684 protein-coding genes (mean exons per gene, 3.85; mean gene length, 2638 bp; and mean complete coding sequence length, 1669 bp; Table S7). More than 91.67% of the identified genes were functionally annotated via searches of the NCBI non-redundant protein, SwissProt, and KEGG databases. This reduced quantity of genes, compared to the 16,145 genes in the prior draft genome, indicates that the updated assembly contains fewer contaminant and fragmented genes (Table S8). Through integration of all predicted repeat results, about 9.7% of the genome could be attributed to transposable elements (TEs) and the highest content of family was DNA (Tables S9 and S10). We updated the mitochondrial genome assembly, which was found to encode 37 genes, including 13 protein-coding genes, 2 rRNA genes, and 22 tRNA genes (Fig. 1B).

Assessment of the quality of our assembly and annotation in BUSCO (Benchmarking Universal Single-Copy Orthologs) indicated that our current Nanopore and prior NGS assemblies were 96.70% and 93.40% complete, respectively. Gene set completeness levels were 98.40% and 94.60% for the current Nanopore and prior NGS assemblies, respectively, indicating that the new one has 12.60% greater gene completeness than the prior draft. Moreover, the level of gene completeness of the present assembly exceeds that obtained in prior *Arachnida* genome efforts, including those for *Ixodes scapularis* (78.80%), *Stegodyphus mimosarum* (81.20%), and *Tetranychus urticae* (92.40%) (Table S11).

The dramatically improved assembly statistics obtained here relative the original draft genome is consequential because high-quality transcript data are conducive to allergen gene discovery. Our cloned Der f 23 cDNA sequence is same as the Der f 23 sequence in the assembled transcriptome, but different from that in the former draft genome.^13^ This improvement can be attributed to our combined use of multiple sequencing methods with complementary technical advantages that facilitated the rapid accurate *de novo* assembly (Fig. S2).^15^

As of October 2019, 959 allergens had been collected in the WHO/IUIS allergen database. We used homology analysis to align our assembled transcriptome data with amino acid sequences in the WHO/IUIS database. With a BLAST filter of identify ≥50%, 29 homologs were filtered out (Table S12). The first 8 candidate homologs of interest were cloned and expressed for identification of allergenicity (Table 1). The results showed that petrotrophic-like protein in Der f (Genbank No. MK419030.1) has 74.90% homology with Der p 37 allergen gene (Genbank No. AVD73319.1). We obtained positive IgE-binding results with specific IgEs from the serum of individuals with strong HDM allergies (sIgE) for Der f petrotrophic-like protein [positive rate: 21.4%, 41/192 in IgE-enzyme-linked immunosorbent assay (ELISA); 4/10 in IgE-western blot (WB); 4/10 in IgE-dot ELISA] (Fig. 1C-D and Fig. S3). Troponin C-like protein from Der f (Genbank No. MK419032.1) was found to have 95.42% homology with the allergen Tyr p 34 (Genbank No. ACL36923.1), and a positive sIgE-binding reaction (positive rate: 9.21%, 7/76 in IgE-ELISA; positive serum: 7/7 in IgE-WB; 6/7 in IgE-dot ELISA) (Fig. 1E-F and Fig. S4). Based on these results affirming that these 2 homologs are novel Der f allergens, they have been named Der f 37 and Der f 39 by WHO/IUIS, respectively (Table 1). We did not observe sIgE binding for the remaining 6 homologs, and thus can infer they are unlikely to be allergens (Table 1). Finally, we retrieved complete gene sequences and genomic location of 33 canonical HDM allergens and 2 novel HDM allergens encoded in the assembled *D. farinae* genome (Table S13).

**Table 1:** Identification of HDM-specific IgE binding activity of 8 allergen homologs in the *D. farinae* genome.

Locus tag (GenBank No.)	Biochemical function	Deduced no. amino acids	Homolog (GenBank No.) % similarity species	Allergenicity	WHO/IUIS Allergen Nomenclature^b^
Dfarinae12240 (MK419032.1)	Troponin C-like protein	153	Tyr p 34 (ACL36923.1) 95.42% *Tyrophagus putrescentiae*	IgE-ELISA 9.21% (7/76); IgE-WB^a^ 100% (7/7); IgE-dot blot^a^ 85.71% (6/7)	Der f 39
Dfarinae12320 (MT360915.1)	Heat shock cognate 70-like protein	202	Aed a 8 (ABF18258.1) 84.83% *Aedes aegypti*	No activity	None
Dfarinae07901 (MT360919.1)	Bacterial lytic enzyme-like protein	150	Der p 38 (AAN02509.1) 80.00% *Dermatophagoides pteronyssinus*	No activity	None
Dfarinae06505 (MK419030.1)	Petrotrophic-like protein	250	Der p 37 (AVD73319.1) 74.90% *Dermatophagoides pteronyssinus*	IgE-ELISA 21.4% (41/192); IgE-WB 40% (4/10); IgE-dot blot 40% (4/10)	Der f 37
Dfarinae09175 (MT360914.1)	Cytochrome *c*- like protein	106	Cur l 3 (AAK67492.1) 66.99% *Curvularia lunata*	No activity	None
Dfarinae11953 (MT360916.1)	Peptidyl-prolyl *cis*-trans isomerase-like protein	227	Asp f 27 (CAI78448.1) 59.15% *Aspergillus fumigatus*	No activity	None
Dfarinae11869 (MT360917.1)	Lysosomal aspartic protease-like protein	401	Aed a 11 (XP_001657556.1) 54.36% *Aedes aegypti*	No activity	None
Dfarinae12442 (MT360918.1)	Aldehyde dehydrogenase-like protein	490	Cla h 10 (CAA55072.2) 50.63% *Cladosporium herbarum*	No activity	None

a HDM allergic sera from positive samples in IgE-ELISA assay.

b WHO/IUIS database at http://www.allergen.org/)./, not available; BLAST, Basic Local Alignment Search Tool, https://blast.ncbi.nlm.nih.gov/Blast.cgi

Allergen homologs, including homologs of panallergens, can be considered potential allergen candidates.^16^ We obtained 6 candidates with amino acid sequence homologies to canonical allergens ranging from 50.63% to 84.83% (Table 1). These candidates are: heat shock cognate 70-like protein (GenBank No. MT360915.1), homolog of Aed a 8; bacterial lytic enzyme-like protein (GenBank No. MT360919.1), homolog of Der p 38; cytochrome c-like protein (GenBank No. MT360914.1), homolog of Cur l 3; peptidyl-prolyl *cis*-trans isomerase-like protein (GenBank No. MT360916.1), homolog of Asp f 27; lysosomal aspartic protease like protein (GenBank No. MT360917.1), homolog of Aed a 11; and aldehyde dehydrogenase-like protein (GenBank No. MT360918.1), homolog of Cla h 10. These 6 homologs exhibited no binding activity with sIgE (N = 15; >100 kU_A_/L), nor with IgEs from non-HDM allergic individuals (N = 10) (Figs. S5, S6). To further confirm whether the Der p 38 homolog bacterial lytic enzyme-like protein has sIgE binding activity, an additional sIgE binding assay was conducted with an expanded sample of HDM allergic sera (N = 100). Similarly, an *Escherichia coli*-derived recombinant protein of the Der p 38 homolog showed no sIgE-binding activity (N = 100) (Fig. S6). Der p 38 (GenBank No. MT360919.1) differs from Der f 38 (GenBank No. QHQ72282.1) by 2 amino acids (Fig. S7). Both have yielded positive skin prick test results and thus were recognized as allergens by WHO/IUIS despite there being no published IgE-binding assays (Der p 38: http://www.allergen.org/viewallergen.php?aid=949 and Der f 38: http://www.allergen.org/viewallergen.php?aid=1014). The remaining 21 additional proteins with ≥50% homologous amino acid sequence require further allergenicity probing (Table S12).

In summary, we used multiple sequencing technologies to assemble a Der f chromosome-level genome and transcriptome. We identified 2 previously unknown allergens: Der f 37 and Der f 39. These findings will be helpful for clarifying pathogenic mechanisms of HDM allergies and for supporting the development of diagnostic methods and immunotherapeutic vaccines.

## Abbreviations

House dust mites (HDMs); *Dermatophagoides pteronyssinus* (Der p); *Dermatophagoides farinae* (Der f); Next generation sequencing (NGS); World Health Organization/International Union of Immunological Societies Allergen Nomenclature Sub-committee (WHO/IUIS).

## Funding

The present study was supported in part by research funding from the 10.13039/501100001809National Natural Science Foundation of China (Grants No. 81571570, 82071806, 30671943 and 30671943), 10.13039/501100009019SZU Top Ranking Project (Grant No. 86000000210) and Shenzhen City (Grants No. JSGG20200102165803939 and JSGG20200225151806035).

## Authors’ consent for publication

All the authors consent the publication of the manuscript.

## Author contributions

JC, ZC, JH, YBH, YSH and ZZ performed experiments and interpreted results. JC, YH, ZZ, PG, WZ, DF and JL contributed to the data analysis. JC, JS, and KJ supervised the projects and participated in experimental design and technical discussions. JC, DF and ZZ wrote the paper. ZFZ, JS, JL and KJ revised the manuscript.

## Availability of data and materials

The datasets used and/or analysed during the current study are available from the corresponding author on reasonable request.

## Ethics approval and consent to participate

Permission to conduct this study was obtained from the Ethics Committee of the First Affiliated Hospital of Guangzhou Medical College (No. 2012-51). Informed consent was obtained from all individual participants included in the study. All procedures involving human participants were in accordance with the ethical standards of the committee.

## Declaration of competing interest

The authors declare no competing interests.

## Appendix ASupplementary data

**Fig. S1**. Microbial content proportion of *D. farinae* egg genome. Microscope observation of egg with low (100 × ; **A**) and high (400 × ; **B**) magnification. Comparison of prokaryotic read content (**C**) and species (**D**) between egg- and body-derived genome sequence reads. Comparison of eukaryotic reads content (**E**) and species (**F**) between dust mite eggs and bodies genome sequence reads.

**Fig. S2**. Pipelines of hybrid assembly of short and long reads for *D. farinae* genome assembly, annotation and evaluation. **A.** Flow chart of genome assembly and annotation of *D. farinae*. **B.** Flow chart of *D. farinae* genome evaluation.

**Fig. S3**. Identification of Der f 37 as a novel HDM allergen. **A.** Deduced amino acid sequence of petrotrophic-like protein (GenBank accession no. MK419030). Synthesized Der f 37 cDNA was subcloned into pET expression system to generate Der f 37 (theoretical molecular mass: ∼36 kDa). **B.** Sodium dodecyl sulfate polyacrylamide gel electrophoresis (SDS-PAGE) of purified rDer f 37 from (a) *E. coli* BL21 cells with lambda CE6 infection or (b) BL21(DE3) cells stained with Coomassie brilliant blue. **C.** Dot blot assay identifying rDer f 37 protein binding by IgE in sera from 10/10 patients with HDM allergies and 0/10 non-HDM allergic subjects (control).

**Fig. S4**. Identification of Der f 39 as a novel HDM allergen. **A.** Deduced amino acid sequence of troponin C like protein (GenBank accession no. MK419032). Synthesized Der f 39 cDNA was subcloned into pET expression system to generate Der f 37 (theoretical molecular mass: ∼17 kDa). **B.** SDS-PAGE analysis of purified rDer f39 from *E. coli* BL21(DE3) cells transformed with pET-His-Der f 39 plasmid stained with Coomassie brilliant blue. **C.** dot blot assay identifying rDer f 39 protein binding by IgE in sera from 10/10 patients with HDM allergies and 0/10 non-allergic subjects (control). Der f 1/2 fusion protein was used as a positive control.

**Fig. S5**. Recombinant expression of six candidate allergen homologs in *D. farina.***A.** Cytochrome *c* like protein, GenBank accession no. MT360914. **B.** Aldehyde dehydrogenase like protein, GenBank accession no. MT360918. **C.** Peptidyl-prolyl *cis*-trans isomerase like protein, GenBank accession no. MT360916. **D.** Lysosomal aspartic protease like protein, GenBank accession no. MT360917. **E.** Bacterial lytic enzyme like protein, GenBank accession no. MT360919. **F.** Heat shock cognate 70 like protein, GenBank accession no. MT360915) was obtained with the use of a pET expression system.

**Fig. S6**. IgE binding activity of six candidate allergen homolog recombinant proteins determined by IgE-ELISA with sera from 15 HDM-allergic individuals (all, >100 kU_A_/L) and 10 healthy non-HDM allergic individuals. The Der p 38 allergen homolog recombinant bacterial lytic enzyme like protein did not react with 100 individual HDM-allergic sera.

**Fig. S7**. Amino acid sequence differences between *D. farinae* bacterial lytic enzyme like protein; GenBank no. MT360919, Der f 38) found in this study and its Der p 38 homolog (GenBank no. QHQ72282.1; http://www.allergen.org/viewallergen.php?aid=1014).

**Table S1**. Summary of *D. farinae* genome and transcriptome sequencing dataset characteristics. ∗DNA sequence downloaded from NCBI (BioProject ID: XXX).

**Table S2**. Summary of Nanopore-sequencing statistical data. Raw read means produced in Nanopore platform. Clean read means had adaptors trimmed and had short and low-quality reads removed with Porechop and NanoFilt. Corrected mean trimmed reads were produced in Canu ‘correct’ and ‘trim’ mode to obtain high-quality reads for genome assembly.

**Table S3**. Assembled genome characteristics.

**Table S4**. Contig sequence lengths.

**Table S5**. Comparison of assembly quality between NGS/Hiseq and Nanopore sequence platforms.

**Table S6**. Transcript consensus with isoform sequences determined in Pacbio platform.

**Table S7**. Summary of *D. farinae* gene set characteristics.

**Table S8**. Homology and functional classification of genes annotated in *D. farinae* genome.

**Table S9**. Transposable element (TE) predictions in the *D. farinae* genome.

**Table S10**. Statistics for *D. farinae* genome repeats.

**Table S11**. Assessment of genome assembly completeness in BUSCO.

**Table S12**. Allergen homologs found in the *D. farinae* genome found by searching allergens from other species in the Allergen Online database (≥50% amino acid sequence homology).

**Table S13**. Canonical allergens encoded in the assembled *D. farinae* genome.

## References

[1] Pawankar R., Canonica G.W., Holgate S.T., Lockey R.F., Organization WA (2011).

[2] Voorhorst R., Spieksma F.T.M., Varekamp H., Leupen M.J., Lyklema A.W. (1967). The house-dust mite (*Dermatophagoides pteronyssinus*) and the allergens it produces. Identity with the house-dust allergen. J Allergy.

[3] Miyamoto T., Oshima S., Ishizaki T., Sato S.H. (1968). Allergenic identity between the common floor mite (*Dermatophagoides farinae* Hughes, 1961) and house dust as a causative antigen in bronchial asthma. J Allergy.

[4] Thomas W.R., Smith W.A., Hales B.J., Mills K.L., O'Brien R.M. (2002). Characterization and immunobiology of house dust mite allergens. Int Arch Allergy Immunol.

[5] Chan T.F., Ji K.M., Yim A.K. (2015). The draft genome, transcriptome, and microbiome of *Dermatophagoides farinae* reveal a broad spectrum of dust mite allergens. J Allergy Clin Immunol.

[6] Lin J., Li M., Liu Y. (2015). Expression, purification and characterization of Der f 27, a new allergen from *Dermatophagoides farinae*. Am J Transl Res.

[7] An S., Chen L., Long C. (2013). Dermatophagoides farinae allergens diversity identification by proteomics. Mol Cell Proteomics.

[8] Li Y., Wang Y., Ran P., Yang P., Liu Z. (2019). IgE binding activities and in silico epitope prediction of Der f 32 in Dermatophagoides farinae. Immunol Lett.

[9] ElRamlawy K.G., Fujimura T., Baba K., Kim J.W., Kawamoto C., Isobe T. (2016). Der f 34, a Novel Major House Dust Mite Allergen Belonging to a Highly Conserved Rid/YjgF/YER057c/UK114 Family of Imine Deaminases. J Biol Chem.

[10] Fujimura T., Aki T., Isobe T. (2017). Der f 35: an MD-2-like house dust mite allergen that cross-reacts with Der f 2 and Pso o 2. Allergy.

[11] Bordas-Le Floch V., Le Mignon M., Bussières L. (2017). A combined transcriptome and proteome analysis extends the allergome of house dust mite *Dermatophagoides* species. PLoS One.

[12] Pomés A., Davies J.M., Gadermaier G. (2018). WHO IUIS allergen nomenclature sub-committee. WHO/IUIS allergen nomenclature: providing a common language. Mol Immunol.

[13] He Y., Dou C., Su Y. (2019). Identification of Der f 23 as a new major allergen of *Dermatophagoides farinae*. Mol Med Rep.

[14] Merchant S., Wood D.E., Salzberg S.L. (2014). Unexpected cross-species contamination in genome sequencing projects. PeerJ.

[15] Antipov D., Korobeynikov A., McLean J.S., Pevzner P.A. (2016). hybridSPAdes: an algorithm for hybrid assembly of short and long reads. Bioinformatics.

[16] Chruszcz M., Kapingidza A.B., Dolamore C., Kowal K. (2018). A robust method for the estimation and visualization of IgE cross-reactivity likelihood between allergens belonging to the same protein family. PLoS One.

