# Supplementary material for: Chromosome-level assembly of Dermatophagoides farinae genome and transcriptome reveals two novel allergens Der f 37 and Der f 39
Source: World Allergy Organ J. 2021 Sep 28;14(10):100590. doi: 10.1016/j.waojou.2021.100590 (PMC8487952; doi:10.1016/j.waojou.2021.100590)
Supplement: Multimedia component 1 [file mmc1.pdf]

Supplementary Informations

Chromosome-level assembly of *Dermatophagoides farinae* genome and transcriptome reveals two novel allergens Der f 37 and Der f 39

Table of Contents:

1. Supplementary materials and methods

2. Supplementary figure legends

FIGURE S1.
FIGURE S2.
FIGURE S3.
FIGURE S4.
FIGURE S5.
FIGURE S6.
FIGURE S7.

3. Supplementary table legends

TABLE S1.
TABLE S2.
TABLE S3.
TABLE S4.
TABLE S5.
TABLE S6.
TABLE S7.
TABLE S8.
TABLE S9.
TABLE S10.
TABLE S11.
TABLE S12.
TABLE S13.

1. Supplementary materials and methods

Mite culture and egg isolation

D. farinae culture and purity check were performed as in our previous study [E1]. The purified mite culture mixture including bodies and eggs was dissolved with 1× phosphate buffered saline and then combined 1:1 with a density gradient solution (Histopaque®-1119, Sigma) and centrifuged at 4000 rpm for 5 min. After centrifugation, the solution was divided into three layers, with eggs in the middle liquid layer. The collected and purified eggs were subjected to DNA genome extraction.

Genomic DNA sample preparation and sequencing

Genomic DNA was extracted from HDM whole bodies or eggs and submitted to quality checks as described previously [E1]. For mite-egg genomic-DNA sequencing, NGS was performed on an Illumina HiSeq Xten platform and the library was prepared with 500-bp DNA fragment insertions. In total, 6.55 G bases were generated with pair-end 150 bp. For mite whole body genomic DNA, long-read sequencing was performed on a Nanopore Grid ION X5 platform (Oxford Nanopore Technologies) for two flow cells (R9.4) with 1D ligation library preparation techniques; a total of 25.7 Gb of raw data were collected.

RNA sample preparation and sequencing

RNA was extracted from HDM whole bodies and submitted to quality checks as in our previous study [E1]. *D. farinae* cDNAs were sequenced with Illumina HiSeqXten and PacBio Iso-Seq for transcriptome analysis. A total of 10.66 Gb of paired-end sequencing data was produced in Illumina HiSeqXten (insert size, ~300 bp), and 9.22 Gb of data was generated from PacBio Iso-Seq three libraries (insert sizes: 1 kbp, 2–3 kbp, and 3–6 kbp, respectively).

Microbial content proportion analysis between body and egg sequence reads

To analyze the microbial content proportion in DNA sequence reads, we selected 1

million reads randomly for mite bodies and eggs. Those reads were aligned to nucleotide of GenBank (2018.1) in BLAST2 (blastn -a 40 -e 1e-4 -m 7) [E2]. We assigned read origin based on the best hit with the highest alignment score. Using accession numbers of assigned reads, we converted NCBI taxonomy dump into lineages in Ncbitax2lin (https://github.com/zyxue/ncbitax2lin). Read assignments were classified as *prokaryote* or *eukaryota*.

D. farinae genome assembly

Raw reads with adaptor sequences were trimmed in Porechop v0.2.2 (https://github.com/rrwick/Porechop). Reads that were < 10 kbp with average phred score <8 were removed by NanoFilt v2.0.02 (https://github.com/wdecoster/nanofilt). Filtered reads (22.7 Gb) were inputted into Canu v.1.7.03 for self-correcting, yielding ~8.3Gb of trimmed data [E3]. Canu v.1.7.03 parameters were set as follows: min read length = 10,000; corOutCoverage = 150; and genome size = 60 m. We select 13.1-fold coverage with length > 50 kbp. The wtdbg v1.2.8 assembler was set as follows: -edge-min 2 --rescue-low-cov-edges -p 19 -S 2 --aln-noskip to obtain an assembly.

To remove contamination and redundancy, the initial genome assembly sequences were first aligned by blastn[2] to nucleotide databases of Genbank (E-value $\leq 1e^{-5}$). Then, we checked carefully whether the genome sequences aligned to bacterial sequences or not [E2]. We aligned the NGS sequences with a sequence depth <10, calculated by egg sequence because eggs have less bacterial contamination, to protein databases of Genbank using blastx (E-value $\leq 1e^{-3}$) to examine which species matched [E2]. All matched species were eukaryote, proving that there was no contamination. At this step, we removed nine polluted sequences leaving 1,734,897 bp in total. Sequences shorter than 1 Mbp were aligned to the whole primary assembly in blat v. 35 [E4] with an identity criterion of ≥90%. There were no big gaps. Sequence average depth was half of the genome sequence coverage as hybrid sequences. At this step, we removed six hybrid sequences, leaving 104,483 bp.

Finally, the clean Nanopore reads longer than 50 kb were used to construct a scaffold in SSPACE-LongRead v1-1 with the following parameters: -i 85 -l 5 -o 5000

[E5]. Pbjelly v15.8.24 was used to fill gaps and further splice with the following parameters: -minMatch 8 -minPctIdentity 85 -bestn 1 -nCandidates 20 -maxScore -500 -nproc 32 -noSplitSubreads [E6]. Additionally, LR_Gapcloser was employed to process gap close and result in no scaffold gap [E7]. The longest 100X Nanopore clean reads were aligned to the scaffold with minimap2 v2.12-r836-dirty, and racon v1.3.1 (https://github.com/isovic/racon) was used to polish the scaffold five times iteratively [E8]. Illumina NGS clean reads from eggs were aligned to the scaffold with bwa v 0.7.17-r1188 [E9]. After removal of duplicated reads from polymerase chain reactions with Picard 1.114 (PICARD/MarkDuplicates.jar, https://broadinstitute.github.io/picard/), the bwa alignment results were re-aligned with GATK 3.4-46 IndelRealigner (https://gatk.broadinstitute.org/hc/en-us). Lastly, the scaffolds were polished with pilon v1.22 (https://github.com/broadinstitute/pilon) three times iteratively to obtain the final assembly [E10].

D.farinae genome annotation

We performed repetitive element annotation. Tandem repeats were predicted using Tandem Repeats Finder v4.04 (http://tandem.bu.edu/trf/trf.html). Transposable elements (TEs) were identified by homology-based and *de novo* approaches. For the homology-based approach, we first identified known TEs using Repeat Masker v4.0.7 program against Repbase v21.12 databases [E11]. We then used RepeatProtein Mask (http://www.repeatmasker.org) to identify TEs by aligning the genome sequence to the TE protein database [E11]. For the *de novo* method, we constructed a repeat library generated by RepeatScout v1.0.5 [E12] and ltr_finder v1.07 [E13], then RepeatMasker was used to identify the repeat sequences. In total, 9.7% of the genome bases could be attributed to TEs; among them, the highest content family was DNA transposon.

Then, the soft-mask genome assembly based on transposable elements were further used for gene prediction. The final gene prediction was integrated from three methodologies: *ab initio*, homology, and transcriptome.

(1) *Ab initio* gene prediction

Ab initio prediction was performed based on the soft-masked genome from transposons.

Four programs (AUGUSTUS v3.2.3, snap v2006-07-28, GeneMark v4.35, and fgenesh from MolQuest package v 2.4.5) were used for prediction.

(2) Gene prediction based on homolog evidence

The protein sequences (ftp://ftp.ensemblgenomes.org/pub/metazoa/release-46) of five related representative species (*Tetranychus urticae, Drosophila melanogaster, Daphnia pulex, Ixodes scapularis,* and *Stegodyphus mimosarum*) and published protein sequences of *D. farinae* were aligned to the assembly using BLAST v2.2.26 (-p tblastn -e 1e-05) [E1]. Alignment and identification of accurately spliced alignments was accomplished in Gene Wise v2.2.0.

(3) Gene prediction from transcriptome evidence

The cDNAs that were generated by Pacbio sequencing were aligned against the assembly genome in PASA (identity ≥ 0.95, coverage ≥ 0.95) (https://pasapipeline.github.io/) to predict the possible gene model. Transcript clean reads generated from Illumina sequence platform were aligned to the improved genome assembly in HISTAT v2.1.0, and transcript assembly was conducted with StringTie v1.3.2.

(4) Final gene sets from three sources of evidence

Gene sets obtained as described above were integrated with Evidence Modeler v1.1.1. For genes supported by only the *ab initio* method, we retained putative genes that had at least one protein domain with an InterPro annotation or that had RNA-seq reads mapped with a fragments per kb per million fragments (FPKM) greater or equal to that calculated by StringTie v1.3.2. Finally, a total of 12,872 genes were obtained. Gene functions were assigned based on best-matched hits to NCBI non-redundant protein (NR), SwissProt, and Kyoto Encyclopedia of Genes and Genomes (KEGG) with BLAST (-p blastp -e 1e-5) [E2]. Gene motifs and domains were identified by InterProScan v 5.27 referencing protein databases. The overlap length of each gene's coding region was calculated based on predicted genes from the above *ab initio*, homology, and transcript methods. All genes having ≥50% overlap by at least one method were retained. According to a Venn diagram analysis of evidence, most genes were derived from homology evidence and transcriptome evidence.

Mitochondrion assembly and annotation

First, blasr v5.1 was used to align the clean Nanopore reads to the *D. farinae* mitochondrial sequence (NC_013184.1) assembled by NGS technology with the following parameters: --minMatch 8 --bestn 10 --noSplitSubreads --advanceExactMatches 1 --nCandidates 1 --maxAnchorsPerPosition 1 --sdpTupleSize 7. Next, the reads which alignment length ≥ 1 kb and identity ≥ 90% were selected for Canu v.1.7.0 [E3] assembly with the parameters set as follows: minReadLength = 1000 minOverlapLength=500; corMhapSensitivity = normal; corMinCoverage = 4; corOutCoverage = 40; correctedErrorRate = 0.144; corMaxEvidenceCoverageGlobal = 1.0×; corMaxEvidenceCoverageLocal = 2.0×; corPartitions = 30; ovsMethod = sequential trimReadsCoverage = 1. Finally, the initial assembly generated by canu was polished by pilon v 1.22 three times iteratively to obtain a 14, 736 bp assembly. Protein-coding genes and rRNAs were identified with reference to MITOS (http://mitos.bioinf.uni-leipzig.de/index.py) using the invertebrate genetic code. tRNAs were identified by sequence similarity to *D. farinae* mitochondrion genes (NC_013184.1) by BLAST (-p blastn -e 1e-10).

Analysis of genome assembly completeness

Nanopore clean reads ≥ 50 kb were aligned to the assembly with Graphmap v 0.5.2 and visualized with Tablet. We checked read coverage of the assembly manually [E14], and found that all reads aligned properly and continuously, affirming the accuracy of assembly. BUSCO (version 3) has been widely used for the assessment of genome assemblies and gene sets based on evolutionarily informed expectations of gene content [E15]. To evaluate the completeness of the *D. farinae* genome, BUSCO v 3.0.2 was used in the genome mode and protein mode with eukaryota_odb9 ortholog gene sets.

Homologous alignment of amino acid sequences with allergens named by WHO/IUIS

As of October 2019, the allergen online database of the WHO/IUIS has collected 959

allergens (http://www.allergen.org/). To identify the allergen genes on our assembled HDM genome, BLAST (-p tblastn -e 1e-3) in-house and alignment coverage were calculated [E3]. We chose homologous genes with amino acid sequences identity ≥ 50% as potential allergen homologs.

Expression of eight potential allergen homolog genes, protein purification, and IgE-binding assay

We found eight potential allergen homolog cDNAs (encoding troponin C-like protein, heat shock cognate 70-like protein, bacterial lytic enzyme-like protein, petrotrophic-like protein, cytochrome c-like protein, peptidyl-prolyl cis-trans isomerase-like protein, lysosomal aspartic protease-like protein, and aldehyde dehydrogenase-like protein) in *D. farinae* that were present in HDM cDNA libraries; their cDNA sequences matched the results of our transcriptome data, verified by Sanger DNA sequencing. The corresponding amino acid sequences were reported in GenBank (Accession nos. MK419032, MT360915, MT360919, MK419030, MT360914, MT360916, MT360917, and MT360918).

These eight recombinant proteins were expressed and purified according to our protocol [E1, E16, E17]. The recombinant expression plasmids of troponin C-like protein, heat shock cognate 70-like protein, petrotrophic-like protein, lysosomal aspartic protease-like protein, and aldehyde dehydrogenase-like protein were constructed with pET-His vectors. The recombinant expression plasmids of bacterial lytic enzyme-like protein, cytochrome c-like protein, and peptidyl-prolyl cis-trans isomerase like-protein were constructed with pET-DsbA vectors. The purified recombinant proteins were subjected IgE-binding assays (western blots, dot blots, and ELISAs) according to our protocol [E1, E16, E17]. All procedures involving human participants were conducted in accordance with ethical standards. Ethics approval was obtained from the First Affiliated Hospital of Guangzhou Medical College. For statistical analysis, non-normally distributed data were compared between groups with Wilcoxon's rank-sum test (significance at $p < 0.05$). SAS 9.1 for Windows software (SAS Institute, Cary, North Carolina, USA) was used for all analyses.

References

E1. Chan TF, Ji KM, Yim AK, Liu XY, Zhou JW, Li RQ, et al. The draft genome, transcriptome, and microbiome of *Dermatophagoides farinae* reveal a broad spectrum of dust mite allergens. J Allergy Clin Immunol 2015; 135:539-48.

E2. Mount DW. Using the Basic Local Alignment Search Tool (BLAST). CSH Protoc 2007; 2007:pdb. top17.

E3. Koren S, Walenz BP, Berlin K, Miller JR, Bergman NH, Phillippy AM. Canu: scalable and accurate long-read assembly via adaptive k-mer weighting and repeat separation. Genome Res. 2017; 27(5): 722-736.

E4. Kent WJ. BLAT--the BLAST-like alignment tool. Genome Res. 2002; 12(4): 656-64.

E5. Boetzer M, Pirovano W. SSPACE-LongRead: scaffolding bacterial draft genomes using long read sequence information. BMC Bioinformatics. 2014; 15:211.

E6. English AC, Richards S, Han Y, Wang M, Vee V, Qu J, Qin X, Muzny DM, Reid JG, Worley KC, Gibbs RA. Mind the gap: upgrading genomes with Pacific Biosciences RS long-read sequencing technology. PLoS One. 2012; 7(11): e47768.

E7. Xu GC, Xu TJ, Zhu R, Zhang Y, Li SQ, Wang HW, Li JT. LR_Gapcloser: a tiling path-based gap closer that uses long reads to complete genome assembly. Gigascience. 2019; 8(1):giy157.

E8. Li H. Minimap2: pairwise alignment for nucleotide sequences. Bioinformatics. 2018; 34(18):3094-3100.

E9. Li H, Durbin R. Fast and accurate long-read alignment with Burrows-Wheeler transform. Bioinformatics. 2010; 26(5):589-95.

E10. Walker BJ, Abeel T, Shea T, Priest M, Abouelliel A, Sakthikumar S, et al. Pilon: an integrated tool for comprehensive microbial variant detection and genome assembly improvement. PLoS One. 2014; 9(11): e112963.

E11. Bao W, Kojima KK, Kohany O. Repbase Update, a database of repetitive elements in eukaryotic genomes. Mob DNA. 2015; 6:11.

E12. Price AL, Jones NC, Pevzner PA. De novo identification of repeat families in large genomes. Bioinformatics. 2005; 21 Suppl 1:i351-8.

E13. Xu Z, Wang H. LTR_FINDER: an efficient tool for the prediction of full-length LTR retrotransposons. Nucleic Acids Res. 2007; 35(Web Server issue): W265-8.

E14. Milne I, Stephen G, Bayer M, Cock PJ, Pritchard L, Cardle L, et al. Using Tablet for visual exploration of second-generation sequencing data. Brief Bioinform. 2013; 14(2):193-202

E15. Waterhouse RM, Seppey M, Simão FA, Manni M, Ioannidis P, Klioutchnikov G, et al. BUSCO Applications from Quality Assessments to Gene Prediction and Phylogenomics. Mol Biol Evol. 2018; 35(3):543-548.

E16. Zhang Z, Cai Z, Hou Y, Hu J, He Y, Chen J, et al. Enhanced sensitivity of capture IgE-ELISA based on a recombinant Der f 1/2 fusion protein for the detection of IgE antibodies targeting house dust mite allergens. Mol Med Rep. 2019 May;19(5):3497-

3504.
E17. He Y, Dou C, Su Y, Chen J, Zhang Z, Zhao Z, et al. Identification of Der f 23 as a new major allergen of *Dermatophagoides farinae*. Mol Med Rep. 2019; 20(2):1270-1278.

2. Supplementary figure legends

FIGURE S1. Microbial content proportion of *D. farinae* egg genome. Microscope observation of egg with low (100×; **A**) and high (400×; **B**) magnification. Comparison of prokaryotic read content (**C**) and species (**D**) between egg- and body-derived genome sequence reads. Comparison of eukaryotic reads content (**E**) and species (**F**) between dust mite eggs and bodies genome sequence reads.

FIGURE S2. Pipelines of hybrid assembly of short and long reads for *D. farinae* genome assembly, annotation and evaluation. **A.** Flow chart of genome assembly and annotation of *D. farinae*. **B.** Flow chart of *D. farinae* genome evaluation.

FIGURE S3. Identification of Der f 37 as a novel HDM allergen. **A.** Deduced amino acid sequence of petrotrophic-like protein (GenBank accession no. MK419030). Synthesized Der f 37 cDNA was subcloned into pET expression system to generate Der f 37 (theoretical molecular mass: ~36 kDa). **B.** Sodium dodecyl sulfate polyacrylamide gel electrophoresis (SDS-PAGE) of purified rDer f 37 from (a) *E. coli* BL21 cells with lambda CE6 infection or (b) BL21(DE3) cells stained with Coomassie brilliant blue. **C.** IgE binding activity determined by IgE-western blots of rDsbA-Der f37 ($Z = 5.198$, $p < 0.01$) with individual sera from 192 HDM-allergic patients and 20 healthy non-allergic individuals. Western blot **(D)** and dot blot assay **(E)** identifying rDer f 37 protein binding by IgE in sera from 10/10 patients with HDM allergies and 0/10 non-HDM allergic subjects (control).

FIGURE S4. Identification of Der f 39 as a novel HDM allergen. **A.** Deduced amino acid sequence of troponin C like protein (GenBank accession no. MK419032). Synthesized Der f 39 cDNA was subcloned into pET expression system to generate Der f 37 (theoretical molecular mass: ~17 kDa). **B.** SDS-PAGE analysis of purified rDer f39 from *E. coli* BL21(DE3) cells transformed with pET-His-Der f39 plasmid stained with Coomassie brilliant blue. **C.** IgE binding activity determined by IgE-ELISA of rDer f39 ($Z = 2.587$, $p < 0.01$) with individual sera from 76 HDM-allergic patients and 20 healthy non-allergic individuals. Western blot **(D)** and dot blot assay **(E)** identifying rDer f 39 protein binding by IgE in sera from 10/10 patients with HDM allergies and

0/10 non-allergic subjects (control). Der f 1/2 fusion protein was used as a positive control.

FIGURE S5. Recombinant expression of six candidate allergen homologs in *D. farina.* **A.** Cytochrome c like protein, GenBank accession no. MT360914. **B.** Aldehyde dehydrogenase like protein, GenBank accession no. MT360918. **C.** Peptidyl-prolyl cis-trans isomerase like protein, GenBank accession no. MT360916. **D.** Lysosomal aspartic protease like protein, GenBank accession no. MT360917. **E.** Bacterial lytic enzyme like protein, GenBank accession no. MT360919. **F.** Heat shock cognate 70 like protein, GenBank accession no. MT360915) was obtained with the use of a pET expression system.

FIGURE S6. IgE binding activity of six candidate allergen homolog recombinant proteins determined by IgE-ELISA with sera from 15 HDM-allergic individuals (all, >100 kU_A/L) and 10 healthy non-HDM allergic individuals. The Der p 38 allergen homolog recombinant bacterial lytic enzyme like protein did not react with 100 individual HDM-allergic sera.

FIGURE S7.

Amino acid sequence differences between *D. farinae* bacterial lytic enzyme like protein; GenBank no. MT360919, Der f 38) found in this study and its Der p 38 homolog (GenBank no. QHQ72282.1; http://www.allergen.org/viewallergen.php?aid=1014).

3. Supplementary table legends

TABLE S1. Summary of *D. farinae* genome and transcriptome sequencing dataset characteristics. *DNA sequence downloaded from NCBI (BioProject ID: XXX).

TABLE S2. Summary of Nanopore-sequencing statistical data. Raw read means produced in Nanopore platform. Clean read means had adaptors trimmed and had short and low-quality reads removed with Porechop and NanoFilt. Corrected mean trimmed reads were produced in Canu 'correct' and 'trim' mode to obtain high-quality reads for genome assembly.

TABLE S3. Assembled genome characteristics.

TABLE S4. Contig sequence lengths.

TABLE S5. Comparison of assembly quality between NGS/Hiseq and Nanopore sequence platforms.

TABLE S6. Transcript consensus with isoform sequences determined in Pacbio platform.

TABLE S7. Summary of *D. farinae* gene set characteristics.

TABLE S8. Homology and functional classification of genes annotated in *D. farinae* genome.

TABLE S9. Transposable element (TE) predictions in the *D. farinae* genome.

TABLE S10. Statistics for *D. farinae* genome repeats.

TABLE S11. Assessment of genome assembly completeness in BUSCO.

TABLE S12. Allergen homologs found in the *D. farinae* genome found by searching allergens from other species in the Allergen Online database (≥50% amino acid sequence homology)

TABLE S13. Canonical allergens encoded in the assembled *D. farinae* genome.

TABLE S1.

Molecule	Platform	Source	Libraries	Bases (Gb)	Genome coverage
DNA	NGS/Hiseq Xten	Egg	1	6.55	115.30
	*NGS/Hiseq Xten	Whole body	1	9.70	170.75
	Nanopore	Whole body	1	25.76	453.47
RNA (cDNA)	NGS/Hiseq Xten	Whole body	1	10.66	187.65
	Pacbio Sequel I	Whole body	3	9.22	162.31

TABLE S2.

Characteristic	Raw	Clean	Corrected and trimmed
**Total length (bp)**	25,759,950,168	22,699,378,819	8,329,616,326
**Genome_coveage**	396	349	128
**Max_length**	134,855	134,803	120,414
**>2 kb**	1,481,407	984,435	246,466
**>5 kb**	1,265,176	984,435	246,466
**>10 kb**	986,475	984,435	246,466
**Nx**	Length	Length	Length
**N90**	9,188	13,547	25,786
**N50**	23,944	26,034	34,426

TABLE S3

Nuclear genome	Value
Assembly length	58.77 Mb
Number of contigs	10
Contig N50	9.27 Mb
Longest Contigs	13.80 Mb
%GC	30.4
Repeat region % of assembly	9.73
Predicted gene models	10,684
Average coding sequence length	1,669.11 bp
Average exons per gene	3.85
**Mitochondrial genome**	
Assembly length	14,736 bp
Protein coding gene number	13
rRNA gene number	2
tRNA gene number	22

TABLE S4.

ID	Length	GC%
**ctg1**	13,798,924	30.11
**ctg2**	9,845,874	30.28
**ctg3**	9,265,486	30.28
**ctg4**	5,189,645	30.54
**ctg5**	4,892,402	30.48
**ctg6**	4,631,692	30.92
**ctg7**	4,592,003	30.57
**ctg8**	3,602,211	30.75
**ctg9**	2,344,723	29.88
**ctg10**	613,882	31.90

TABLE S5.

Feature	NGS/Hiseq				Nanopore	
	scaffold		contig		Contig	
	Length (bp)	n	Length (bp)	n	Length (bp)	n
Max length	778,042		107,957		13,798,924	
N10	481,692	9	25,862	134	13,798,924	1
N20	368,376	22	18,791	373	13,798,924	1
N30	287,549	39	14,148	691	9,845,874	2
N40	235,409	59	11,121	1,104	9,845,874	2
N50	186,342	85	8,538	1,637	9,265,486	3
N60	143,844	118	6,673	2,324	5,189,645	4
N70	110,403	161	4,956	3,220	4,892,402	5
N80	81,164	217	3,499	4,456	4,631,692	6
N90	44,152	302	2,071	6,350	3,602,211	8
Total length	53,545,338		51,608,045		58,776,842	
Number ≥ 100 bp	515		11,600		10	
number ≥ 2000 bp	515		6,481		10	
GC%	29.40		30.50		30.40	

Hiseq platform sequences from NCBI accession no. ASGP00000000.

TABLE S6.

Max length	Total number	Average length	Number > 2000 bp	Total length	N50
**8,718**	41,602	2,387	25,374	99,320,767	2,627

TABLE S7.

Source	Number	Average CDS length (bp)	Average exons per gene	Average exon length (bp)	Average intron length (bp)
**Nanopore**	10,684	1,669.11	3.85	433.29	339.89
**Hiseq**	16,145	1,076.38	2.60	414.57	822.30

Nanopore gene set from this project. Hiseq gene set from NCBI accession no. XXX.

TABLE S8.

Gene status		No. genes	Percentage in gene set
**Total**		10,684	
**Annotated**	InterPro	8,002	74.90%
	Swissprot	7,703	72.10%
	KEGG	7,460	69.82%
	GO	6,119	57.27%
	NR	9,671	90.52%
**Unannotated**		890	8.33%

NR, RefSeq non-redundant proteins (ftp://ftp.ncbi.nih.gov/blast/db/FASTA).

TABLE S9.

Method	Repeat size	% of genome
**Trf**	1,274,524	2.17
**Repeatmasker**	3,566,475	6.07
**Proteinmask**	297,587	0.51
De novo	1,922,908	3.27
**Total**	5,718,447	9.73

TABLE S10.

Type	Repbase TEs		TE proteins		*De novo*		Combined TEs	
	Length (bp)	% in genome	Length (bp)	% in genome	Length (bp)	% in genome	Length (bp)	% in genome
**DNA**	2,760,663	4.70	10,805	0.02	39,000	0.07	2,798,041	4.76
**LINE**	688,312	1.17	66,470	0.11	128,157	0.22	854,680	1.45
**SINE**	14,422	0.02	0	0.00	0	0.00	14,422	0.02
**LTR**	817,898	1.39	220,312	0.37	644,566	1.10	1,421,472	2.42
**Other**	611	0.00	0	0.00	0	0.00	611	0.00
**Unknown**	0	0.00	0	0.00	1,141,531	1.94	1,141,531	1.94
**Total**	3,566,475	6.07	297,587	0.51	1,909,345	3.25	5,077,479	8.64

Repbase TEs: RepeatMasker analysis results using Repbase. TE proteins: RepeatProteinMask analysis results using Repbase. *De novo*: RepeatMasker analysis results using library predicted by the *de novo* method. Combined: combined results for Repbase TEs, TE proteins, and the *de novo* method.

TABLE S11.

Type	Species	Platform	BUSCO		
			Complete	Fragmented	Missing
Gene	*Ixodes scapularis*	NGS/Hiseq	78.80%	11.90%	9.30%
	Stegodyphus mimosarum	NGS/Hiseq	81.20%	14.90%	3.90%
	Tetranychus urticae	NGS/Hiseq	92.40%	2.30%	5.30%
	Dermatophagoides farinae	Nanopore	97.40%	1.00%	1.60%
	Dermatophagoides farinae	NGS/Hiseq	84.80%	9.90%	5.30%
Genome	*Dermatophagoides farinae*	Nanopore	96.00%	0.70%	3.30%
	Dermatophagoides farinae	NGS/Hiseq	92.10%	1.30%	6.60%

I. scapularis, S. mimosarum, and *T. urticae* sequences were downloaded from the Ensembl metazoa database. Hiseq platform *D. farina* sequence was downloaded from NCBI (accession no. XXX).

TABLE S12.

No	Locus tag	Biochemical function	Deduced no. amino acids	Homolog allergen (GenBank no.) % similarity species	Notes
1	Dfarinae12240	Troponin C	153	Tyr p 34 (ACL36923) 95.42% *Tyrophagus putrescentiae*	Der f 39 (named in this study)
2	Dfarinae12320	Heat shock cognate 70 protein	202	Aed a 8 (ABF18258.1, 84.83%) *Aedes aegypti*	No IgE-ELISA binding
3	Dfarinae07670	Profilin	130	Tyr p 36 (AOD75399.1) 83.21% Tyrophagus putrescentiae	None tested
4	Dfarinae07901	Bacterial lytic enzyme protein	150	Der p 38 (AAN02509.1) 80.00% Dermatophagoides pteronyssinus	No IgE-ELISA binding
5	Dfarinae06505	Chitin binding protein	250	Der p 37 (AVD73319) 74.90% *Dermatophagoides pteronyssinus*	Der f 37 named in this study
6	Dfarinae10302	Beta-Enolase	433	Sal s 2 (ACH70931) 71.3% *Salmo salar*	None tested
7	Dfarinae01101	L3 ribosomal protein	398	Asp f 23 (AAM43909) 67.77% *Aspergillus fumigatus*	None tested
8	Dfarinae03903	Glucose 6-phosphate isomerase	585	Pan h 11 (AAN02509.1) 67.58% *Pangasianodon hypophthalmus*	None tested

9	Dfarinae10944	Glyceraldehyde-3-phosphate-dehydrogenase	332	Tri a 34<br>(CAZ76054) 67.47%<br>*Triticum aestivum*	None tested
10	Dfarinae09175	Cytochrome c like protein	106	Cur l 3<br>(Q96VP3) 66.99%<br>*Curvularia lunata*	No IgE-ELISA binding
11	Dfarinae09015	Heat shock protein P90	718	Asp f 12<br>(AAB51544) 65.34%<br>*Aspergillus fumigatus*	None tested
12	Dfarinae08265	Aldolase A	361	Pan h 3<br>(XP_026771637.1) 65.00%<br>*Pangasianodon hypophthalmus*	None tested
13	Dfarinae07232	Pyruvate kinase	533	Sal s 9<br>(ACH70965.1) 63.28%<br>*Salmo salar*	None tested
14	Dfarinae12793	Aldehyde dehydrogenase	451	Tyr p 35<br>(AOD75396.1) 62.94%<br>*Tyrophagus putrescentiae*	None tested
15	Dfarinae11953	Peptidyl-prolyl cis-trans isomerase protein	227	Asp f 27<br>(CAI78448) 59.15%<br>*Aspergillus fumigatus*	No IgE-ELISA binding
16	Dfarinae03857	Troponin I	211	Pon l 7<br>(P05547) 57.31%<br>*Pontastacus leptodactylus*	None tested
17	Dfarinae09176	Pyruvate kinase PKM-like	528	Pan h 9<br>(XP_026775867.1) 57.17%<br>*Pangasianodon hypophthalmus*	None tested
18	Dfarinae10217	Eukaryotic translation initiation factor 3 subunit	328	For t 2<br>(ACD65081) 56.13%<br>*Forcipomyia taiwana*	None tested
19	Dfarinae04475	Transaldolase	327	Pen ch 35<br>（ADK27483) 56.04%<br>*Penicillium chrysogenum*	None tested
20	Dfarinae04273	Peptidyl-prolyl isomerase	209	Asp f 11	None tested

				(CAB44442) 55.84% *Aspergillus fumigatus*	
21	Dfarinae05803	manganese superoxide dismutase	231	Pis v 4 (ABR29644) 55.45% *Pistacia vera*	None tested
22	Dfarinae09701	Mitochondrial malate dehydrogenase	343	Mala f 4 (AAD25927) 54.68% *Malassezia furfur*	None tested
23	Dfarinae05230	Myosin, light chain	202	Bla g 8 (ABD47458) 54.6% *Blattella germanica*	None tested
24	Dfarinae11869	Lysosomal aspartic protease protein	401	Aed a 11 (XP_001657556.1) 54.36% *Aedes aegypti*	No IgE-ELISA binding
25	Dfarinae03060	Elongation factor 1	73	Tri a 45 (AKJ77985.1) 52.17% *Triticum aestivum*	None tested
26	Dfarinae02193	Thioredoxin	106	Plo i 2 (CBW45298) 51.96% *Plodia interpunctella*	None tested
27	Dfarinae01264	ML-domain protein	143	Blo t 2 (AAQ73483) 50.69% *Blomia tropicalis*	None tested
28	Dfarinae12442	Aldehyde dehydrogenase protein	490	Cla h 10 (CAA55072) 50.63% *Cladosporium herbarum*	No IgE-ELISA binding
29	Dfarinae04109	Acid ribosomal protein P1	119	Cla h 12 (CAA59463) 50% *Cladosporium herbarum*	None tested

TABLE S13.

Location	Allergen	Locus tag*	Biochemical function	Deduced no. amino acids	WHO/IUIS data† (% similarity)
Contig 1	Der f 1	Dfarinae09850	Cysteine protease	321	BAC53948.1 (100.00%)
	Der f 8	Dfarinae08204	Glutathione S-transferase	217	AGC56215.1 (100.00%)
	Der f 18	Dfarinae08412	Chitin-binding protein	462	AAM19082.1 (100.00%)
Contig 2	Der f 3	Dfarinae05328	Trypsin	258	AAP35076.1 (99.22%)
	Der f 5	Dfarinae04701	Low molecular weight IgE binding protein	132	ABO84970.1 (100.00%)
	Der f 21	Dfarinae04702	SH3 domain-binding glutamic acid-rich protein	136	ABO84964.1 (99.26%)
	Der f 27	Dfarinae03993	Serpin	427	AIO08851.1 (99.77%)
	Der f 33	Dfarinae04694	Alpha-tubulin	461	AIO08861.1 (99.78%)
Contig 3	Der f 2	Dfarinae00310	NPC2 family	146	ABG76194.1 (97.95%)
	Der f 13	Dfarinae00170	Fatty acid binding protein	131	AAP35078.1 (100.00%)
	Der f 14	Dfarinae0323	Apolipophorin	1631	AAM21322.1 (86.34%)
	Der f 25	Dfarinae01490	Triosephosphate isomerase	279	AIO08860.1 (100.00%)
	Der f 28	Dfarinae01557	Heat Shock Protein	654	AIO08848.1 (100.00%)
	Der f 30	Dfarinae00321	Ferritin	166	AGC56219.1 (96.79%)
	Der f 31	Dfarinae01441	Cofilin	148	AIO08870.1 (100.00%)
	Der f 35	Dfarinae01264	Uncharacterized protein	143	BAX34757.1 (100.00%)
Contig 4	Der f 6	Dfarinae12003	Chymotrypsin	279	ABG23667.1 (99.64%)
	Der f 7	Dfarinae11783	Bactericidal permeability-increasing like protein	213	ACK76298.1 (99.53%)
	Der f 10	Dfarinae11789	Tropomyosin	284	ABU97468.2 (100.00%)
	Der f 22	Dfarinae10875	lipid binding protein	155	ABG35122.1 (100.00%)
Contig 5	Der f 4	Dfarinae06737	Alpha-amylase	525	AJF93907.1 (99.81%)
	Der f 37	Dfarinae06505	Chitin binding protein	250	QBF67839.1 (100.00%)
Contig 6	Der f 9	Dfarinae02538	Collagenolytic serine protease	276	AIO08869.1 (98.55%)
	Der f 15	Dfarinae02404	Chitinase	573	AAD52672.1 (96.68%)
	Der f 36	Dfarinae02711	Uncharacterized protein	229	ATI08931.1 (100.00%)
Contig 7	Der f 16	Dfarinae05870	Gelsolin/villin	480	AAM64112.1 (100.00%)
	Der f 23	DfarinaeADD 06015	Peritrophin-like protein	174	ALU66112.1 (98.28%)
	Der f 29	Dfarinae06374	Peptidyl-prolyl cis-trans isomerase (cyclophilin)	306	AAP35065.1 (100.00%)
	Der f 32	Dfarinae05847	Secreted inorganic pyrophosphatase	296	AIO08849.1 (100.00%)
	Der f 34	Dfarinae06301	Enamine/imine deaminase	164	A0A1J1DL12.1 (99.22%)
Contig 8	Der f 39	Dfarinae12240	Troponin C	153	QBF67841.1 (100.00%)

Contig 9	Der f 11	Dfarinae07639	Paramyosin	875	AIO08864.1 (99.89%)
	Der f 20	Dfarinae07890	Arginine kinase	311	AIO08850.1 (99.68%)
	Der f 24	Dfarinae07745	Ubiquinol-cytochrome c reductase binding protein	118	AGI78542.1 (100.00%)
	Der f 26	Dfarinae07770	Myosin alkali light chain	160	ATI08939.1 (93.12%)

*Locus tags and contig number are included in our assembled *D. farinae* genome.

†WHO/IUIS Der f 17 listing has no sequence information. There are no Group 12 or 19 allergens in Der f. The bacterial lytic enzyme like protein (GenBank accession no. MT360919) in Der f (Der p 38 homolog) had no IgE-binding activity and was not confirmed as an allergen in this study. Analyzed relative to the NCBI database by BLAST algorithm; GenBank accession numbers are listed. Der f 9 (AIO08869.1) protein sequence is not listed in WHO/IUIS website. Der f 14 (GenBank accession no. BAA04558) has partial sequence.

Figure S1

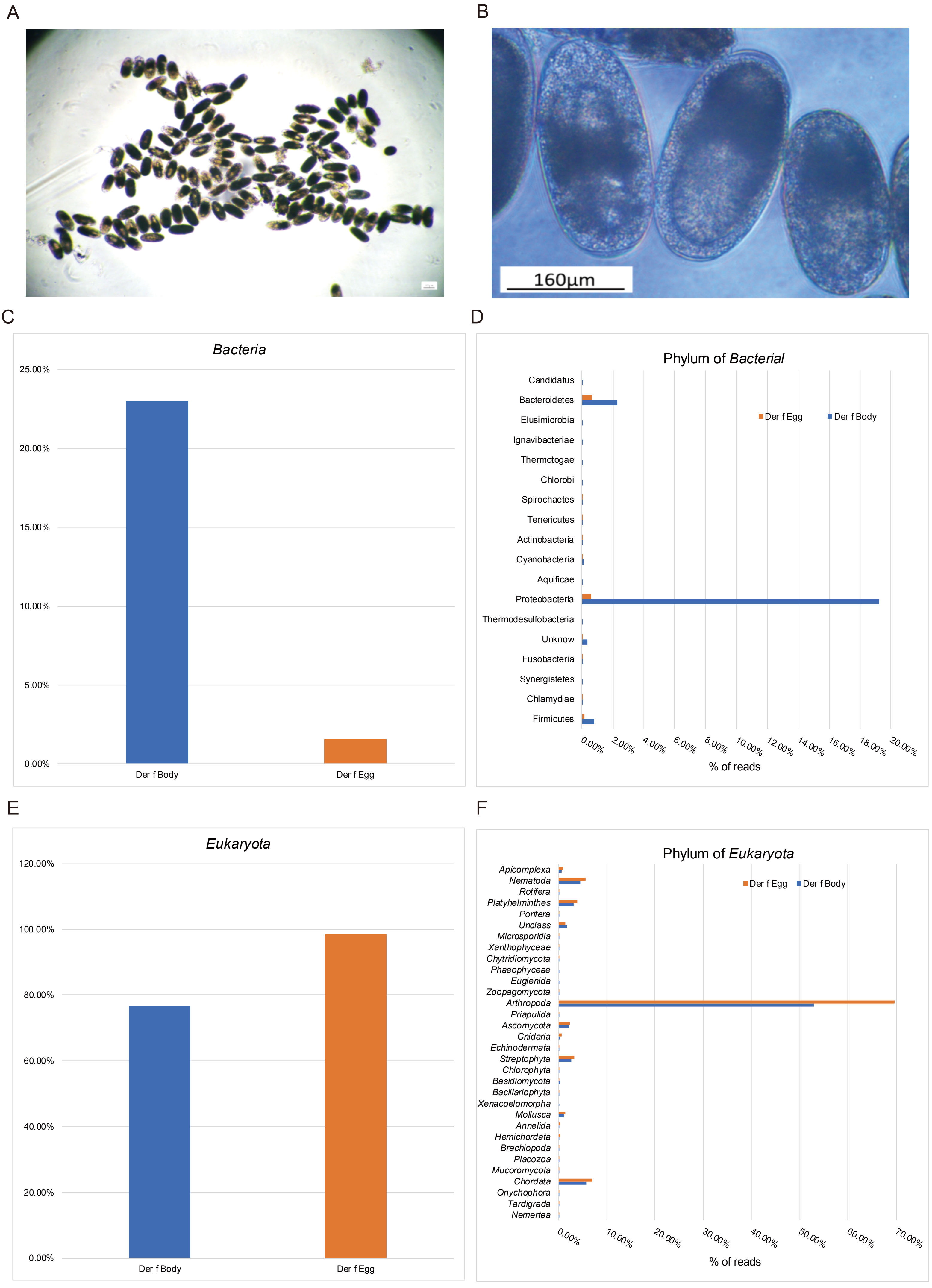
A
B
160μm
C
Bacteria
25.00%
20.00%
15.00%
10.00%
5.00%
0.00%
Der f Body
Der f Egg
D
Phylum of Bacterial
Candidatus
Bacteroidetes
Elusimicrobia
Ignavibacteriae
Thermotogae
Chlorobi
Spirochaetes
Tenericutes
Actinobacteria
Cyanobacteria
Aquificae
Proteobacteria
Thermodesulfobacteria
Unknow
Fusobacteria
Synergistetes
Chlamydiae
Firmicutes
Der f Egg
Der f Body
0.00%
2.00%
4.00%
6.00%
8.00%
10.00%
12.00%
14.00%
16.00%
18.00%
20.00%
% of reads
E
Eukaryota
120.00%
100.00%
80.00%
60.00%
40.00%
20.00%
0.00%
Der f Body
Der f Egg
F
Phylum of Eukaryota
Apicomplexa
Nematoda
Rotifera
Platyhelminthes
Porifera
Unclass
Microsporidia
Xanthophyceae
Chytridiomycota
Phaeophyceae
Euglenida
Zoopagomycota
Arthropoda
Priapulida
Ascomycota
Cnidaria
Echinodermata
Streptophyta
Chlorophyta
Basidiomycota
Bacillariophyta
Xenacoelomorpha
Mollusca
Annelida
Hemichordata
Brachiopoda
Placozoa
Mucoromycota
Chordata
Onychophora
Tardigrada
Nemertea
Der f Egg
Der f Body
0.00%
10.00%
20.00%
30.00%
40.00%
50.00%
60.00%
70.00%
% of reads

Figure S2

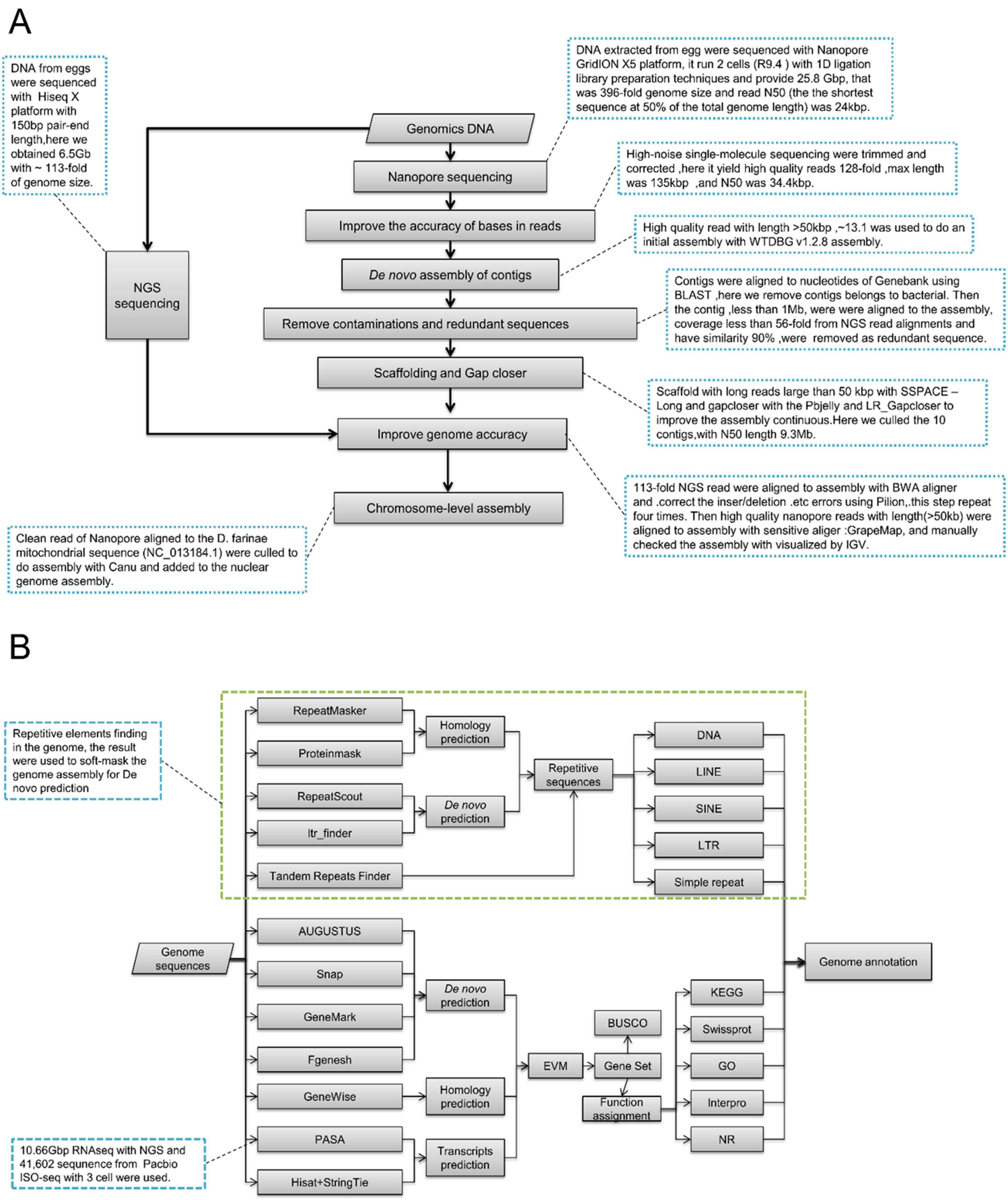
A
DNA from eggs were sequenced with Hiseq X platform with 150bp pair-end length,here we obtained 6.5Gb with ~ 113-fold of genome size.
Genomics DNA
Nanopore sequencing
Improve the accuracy of bases in reads
De novo assembly of contigs
Remove contaminations and redundant sequences
Scaffolding and Gap closer
Improve genome accuracy
Chromosome-level assembly
NGS sequencing
DNA extracted from egg were sequenced with Nanopore GridION X5 platform, it run 2 cells (R9.4) with 1D ligation library preparation techniques and provide 25.8 Gbp, that was 396-fold genome size and read N50 (the the shortest sequence at 50% of the total genome length) was 24kbp.
High-noise single-molecule sequencing were trimmed and corrected ,here it yield high quality reads 128-fold ,max length was 135kbp ,and N50 was 34.4kbp.
High quality read with length >50kbp ,~13.1 was used to do an initial assembly with WTDBG v1.2.8 assembly.
Contigs were aligned to nucleotides of Genebank using BLAST ,here we remove contigs belongs to bacterial. Then the contig ,less than 1Mb, were were aligned to the assembly, coverage less than 56-fold from NGS read alignments and have similarity 90% ,were removed as redundant sequence.
Scaffold with long reads large than 50 kbp with SSPACE – Long and gapcloser with the Pbjelly and LR_Gapcloser to improve the assembly continuous.Here we culled the 10 contigs,with N50 length 9.3Mb.
113-fold NGS read were aligned to assembly with BWA aligner and .correct the inser/deletion .etc errors using Pilion,.this step repeat four times. Then high quality nanopore reads with length(>50kb) were aligned to assembly with sensitive aliger :GrapeMap, and manually checked the assembly with visualized by IGV.
Clean read of Nanopore aligned to the D. farinae mitochondrial sequence (NC_013184.1) were culled to do assembly with Canu and added to the nuclear genome assembly.
B
Repetitive elements finding in the genome, the result were used to soft-mask the genome assembly for De novo prediction
RepeatMasker
Proteinmask
RepeatScout
ltr_finder
Tandem Repeats Finder
Homology prediction
De novo prediction
Repetitive sequences
DNA
LINE
SINE
LTR
Simple repeat
Genome sequences
AUGUSTUS
Snap
GeneMark
Fgenesh
GeneWise
PASA
Hisat+StringTie
De novo prediction
Homology prediction
Transcripts prediction
EVM
BUSCO
Gene Set
Function assignment
KEGG
Swissprot
GO
Interpro
NR
Genome annotation
10.66Gbp RNAseq with NGS and 41,602 sequnence from Pacbio ISO-seq with 3 cell were used.

Figure S3

A

001 MIKFYFLFCSFYIVSVWMLPVSELAGMHQLDEGMINVDDNKPTNLTICVD
051 KKDGTLLGDEHDRTLFYECSNGLAFPFHCPSNLIFDETRGVCIFDPNSPT
101 KRPPNHGNSTYNCTGKEDGFYPDENDATKFHECVNGYPYDFKCPPNTIYD
151 VKRKVCAYKSIERPKEENEQPQPQPQQQHRAVRDVIESTTAEIKEELPT
201 EQSVRRHRRNDEQQQQHHHHQLEEFAKEKNIDLHIIETTTQENFDVWID

B

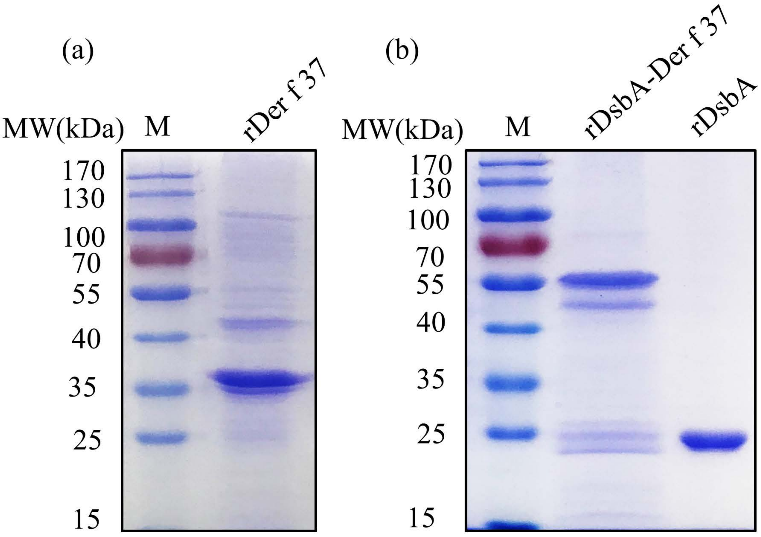

C

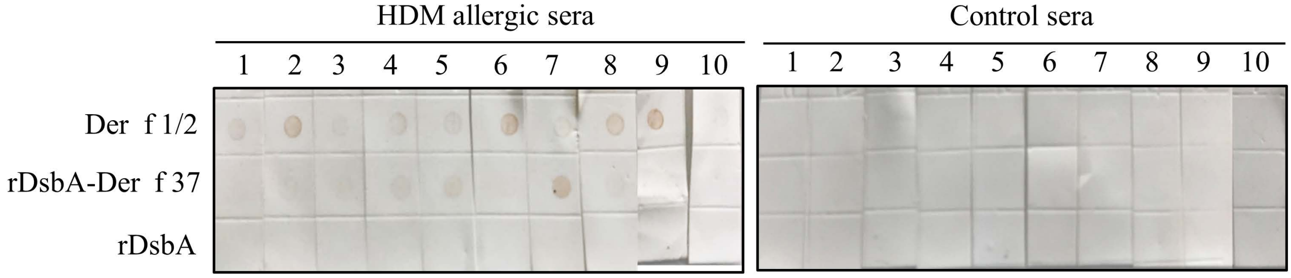

Figure S4

A

001 MSVEELTKEQVQMLRKAFDMFDREKKGHIHTNMVSTILRTLGQTFEEKDL

051 QQLITEIDADGSGELEFDEFLTLTARFLVEEDAEAMQEELREAFRMYDKE

101 GNGYIPTSALREILRALDDKLTNDELDEMIAEIDTDGSGTVDFDEFMEMM

151 TGD

B

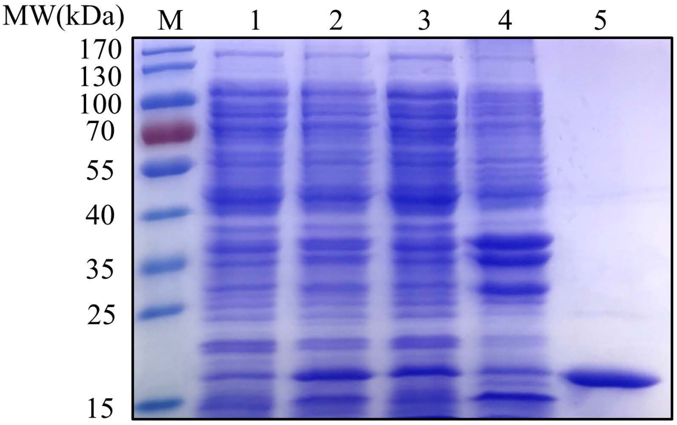

C

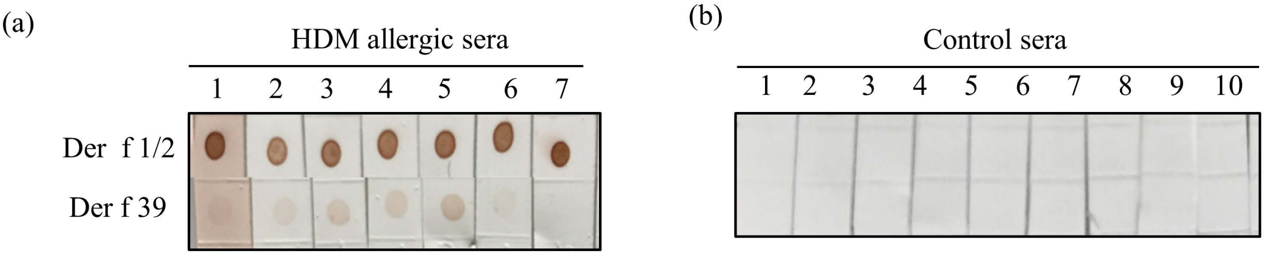

Figure S5

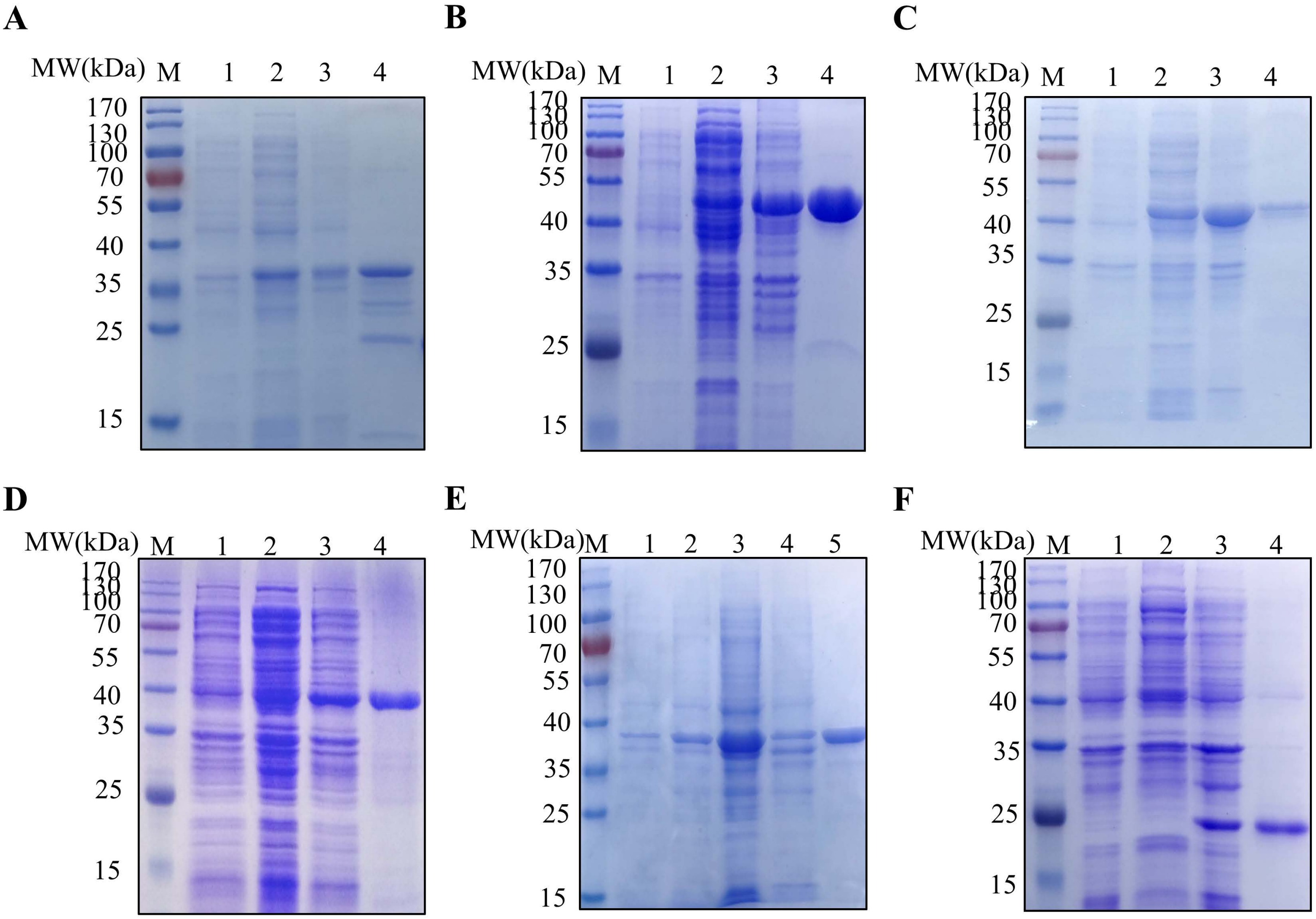
A
MW(kDa) M 1 2 3 4
170
130
100
70
55
40
35
25
15
B
MW(kDa) M 1 2 3 4
170
130
100
70
55
40
35
25
15
C
MW(kDa) M 1 2 3 4
170
130
100
70
55
40
35
25
15
D
MW(kDa) M 1 2 3 4
170
130
100
70
55
40
35
25
15
E
MW(kDa) M 1 2 3 4 5
170
130
100
70
55
40
35
25
15
F
MW(kDa) M 1 2 3 4
170
130
100
70
55
40
35
25
15

Figure S6

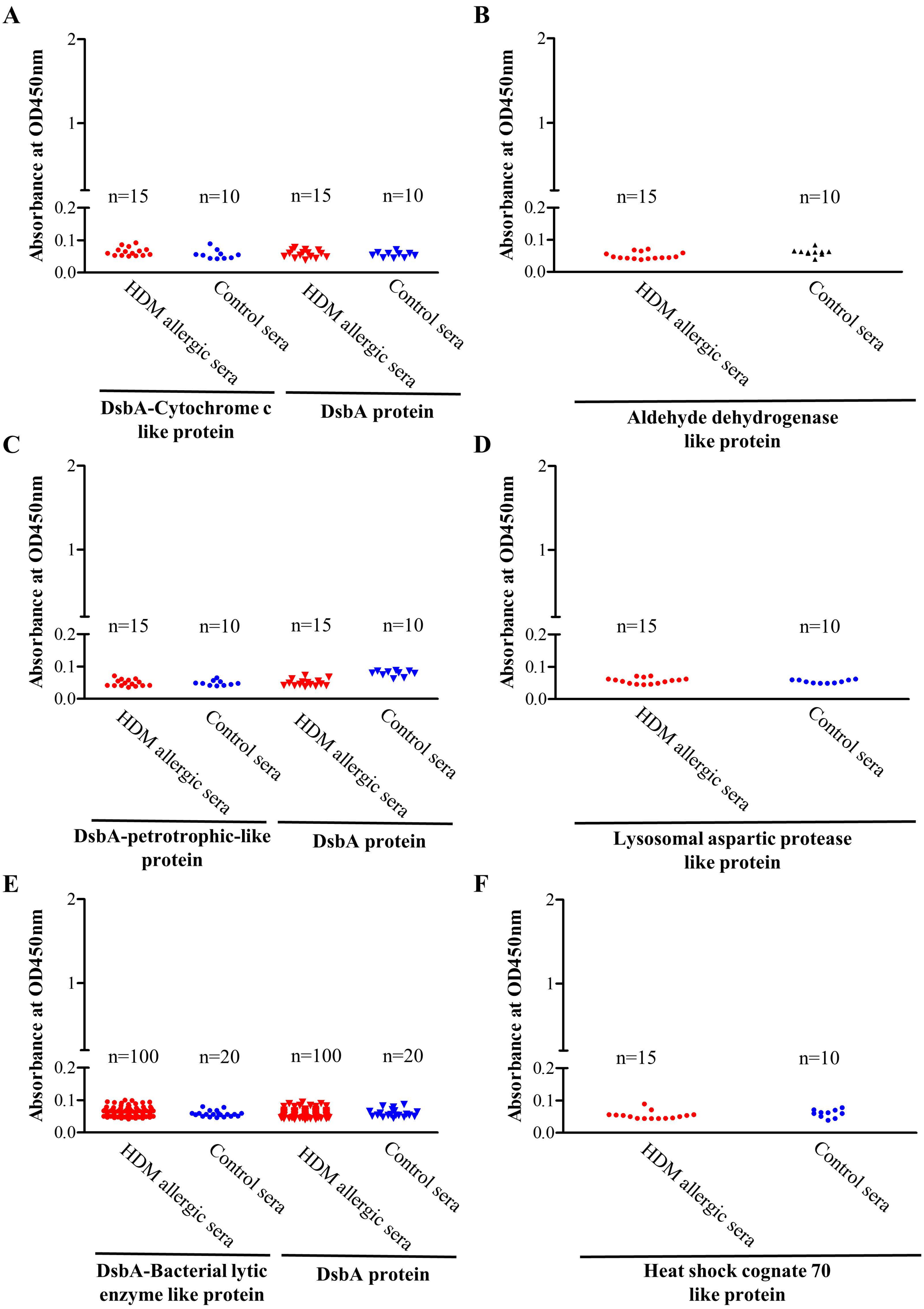

A
B
C
D
E
F
Absorbance at OD450nm
2
1
0.2
0.1
0.0
n=15
n=10
n=100
n=20
HDM allergic sera
Control sera
DsbA-Cytochrome c like protein
DsbA protein
Aldehyde dehydrogenase like protein
DsbA-petrotrophic-like protein
Lysosomal aspartic protease like protein
DsbA-Bacterial lytic enzyme like protein
Heat shock cognate 70 like protein

Figure S7

```
MT360919        MKFFLTLTLFCALAINHVYGDGSHIVKAARSQIGVPYSWGGGGIHGKSKGIGEGANIVGF    60
QHQ72282.1      MKLFLTLTLFCALAISHVYGDGSHIVKAARSQIGVPYSWGGGGIHGKSKGIGEGANIVGF    60
                **:************.********************************************

MT360919        DCSGLAQYSIYQGTHKTIARTAAAQYNDNHCHHVAYGSHQPGDLVFFGNPIYHVGIVSAH    120
QHQ72282.1      DCSGLAQYSIYQGTHKTIARTAAAQYNDNHCHHVAYGSHQPGDLVFFGNPIYHVGIVSAH    120
                ************************************************************

MT360919        GRMVNAPKPGTKVREENIWSYHISHVARCW  150
QHQ72282.1      GRMVNAPKPGTKVREENIWSYHISHVARCW  150
                ******************************
```
